# Diagnosis and treatment of filamentary keratitis in a patient with Demodex infestation––an overlooked risk factor: a case report

**DOI:** 10.1186/s12886-023-02929-x

**Published:** 2023-05-11

**Authors:** Jin Chen, Qinke Yao, Junzhao Chen, Yimin Li

**Affiliations:** 1 Department of Ophthalmology, Ninth People’s Hospital, Shanghai Jiao Tong University School of Medicine, Shanghai, China; 2 Shanghai Key Laboratory of Orbital Diseases and Ocular Oncology, Shanghai, China

**Keywords:** Filamentary keratitis, Demodex infestation, Tea tree oil, Blepharitis, Ocular surface inflammation, Case report

## Abstract

**Background:**

Filamentary keratitis is an ocular condition that is tricky to handle for the difficulty to find the underlying cause. Here we report a case of filamentary keratitis associated with Demodex infestation which highlights the importance of Demodex mites as an easily-overlooked risk factor.

**Case presentation:**

A 63-year-old woman had recurrent symptoms of foreign body sensation and sometimes painful feelings in her left eye soon after her surgical correction of ptosis in this eye. She was then diagnosed as conjunctivitis and given antibiotic eye drops. After one week, the patient complained of aggravation of symptoms with small corneal filaments in the left eye under slit-lamp examination. Despite the removal of filaments and addition of topical corticosteroids and bandage contact lenses, the patient’s condition persisted with enlarged filaments and severe ocular discomfort. 3 days later, eyelashes with cylindrical dandruff were noticed and Demodex infestation was confirmed by microscopic examination of these eyelashes at our clinic this time. She was asked to use tea tree oil lid scrub twice daily. After 3 weeks, her filamentary keratitis was resolved with a dramatic improvement in symptoms and signs. And no recurrence of filamentary keratitis was noticed during the one-year follow-up.

**Conclusions:**

In this case, filamentary keratitis was resolved only with treatment of Demodex infestation while conventional treatment failed. Considering the fact that Demodex infestation is a common but easily overlooked condition, it may be suggestive to take Demodex infestation into account as a risk factor of filamentary keratitis, especially in refractory cases.

## Background

Filamentary keratitis is a chronic ocular condition characterized by formations of filaments which are likely composed of epithelium, mucus and cellular debris on the corneal surface [1]. Symptoms vary from person to person including foreign body sensation, pain, photophobia, hyperemia and lacrimation. Although the pathogenesis remains controversial, filamentary keratitis is seen frequently in certain ocular and systemic conditions, among which dry eye disease ranks the first. Other conditions include immunologic diseases, superior limbic keratoconjunctivitis, exposure keratitis associated with brain stem injury and ocular surgical procedures such as cataract surgery and penetrating keratoplasty.

The management of filamentary keratitis can be clinically tricky, as there are no standard therapies considering the various predisposing factors. Current prevailing management of filamentary keratitis may include removal of filaments, placement of bandage contact lenses, lubrication and anti-inflammatory eye drops, which fail to provide resolution at times. Special management strategies including botulism toxin injection [2], topical hypertonic saline [3] and autologous serum tears [4] are also reported in the literature. However, conventional management does not always work. Seeking the underlying cause of filament generation is of great importance. Here we report a unique case of refractory filamentary keratitis that was resolved on diagnosis and treatment of demodex infestation, which is now believed to be associated with some ocular surface disorders caused by unknown factors.

## Case presentation

**Fig. 1:**
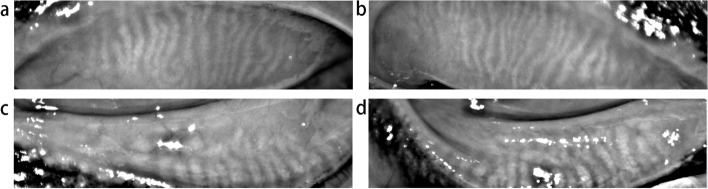
Meibomian glands of both eyes with a little loss and atrophy. **a** upper lid of the left eye. **b** upper lid of the right eye. **c** lower lid of the left eye. **d** lower lid of the right eye

A 63-year-old woman presented with recurrent foreign body sensation, itchiness, conjunctival hyperaemia and sometimes painful eyes in her left eye for 1 year. According to the patient, symptoms actually occurred after a surgical correction of ptosis of the same eye 16 months ago, aggravating gradually for a year before she turned back to her doctor. Her surgery was quite successful and she was able to close her eyes completely, indicating a low possibility of complications such as exposure keratitis due to overcorrection. According to her medical record, a non-invasive tear breakup time and a schirmer test were performed, the results of which were 10 s and 12 mm/5 min in the right eye, while 6 s and 13 mm/5 min in the left eye. She also had her meibomian glands assessed by noncontact infrared meibography, showing few signs of meibomian gland dysfunction (Fig. 1, a-d). She was then diagnosed of conjunctivitis and dry eye and given levofloxacin eye drops and artificial tears.

Two weeks later, the patient complained of aggravation of symptoms. Slit-lamp examination revealed small corneal filaments in her left eye this time. Under topical anesthesia, the filaments were removed by forceps and bandage contact lenses were placed. Tobramycin dexamethasone eye ointment twice daily was also added to the former two drops by her doctor.

**Fig. 2:**
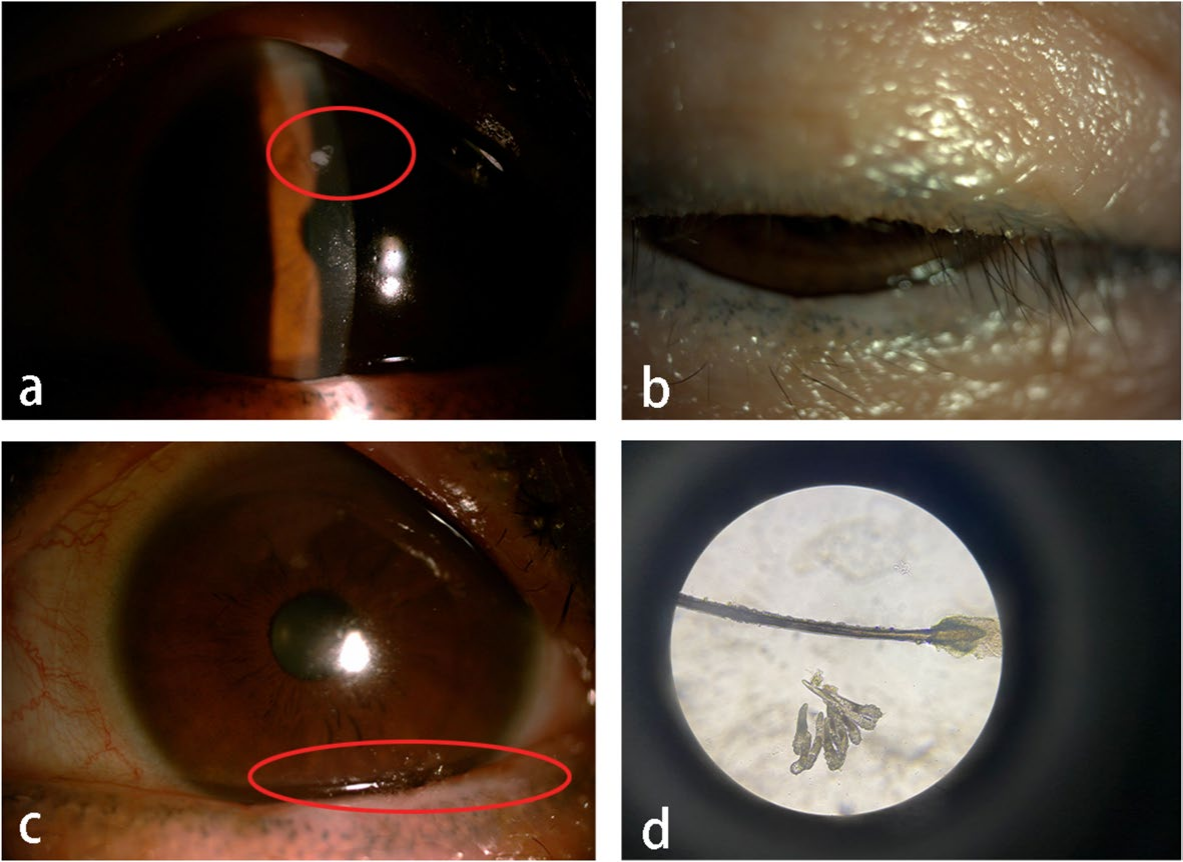
Photographs suggestive of filamentary keratitis and Demodex infestation. **a** filaments remained on the cornea after conventional treatment for 3 weeks, including filaments removal, antibiotic and corticosteroid eyedrops, and bandage contact lense wear(circle). **b** lashes with severe cylindrical dandruff. **c** lid margin hyperemia and foamy tears (circle). **d** severe infestation of Demodex folliculorum and Demodex brevis (magnification 40x)

3 days later, she could not stand the aggravating ocular discomfort and resorted to our clinic this time. Slit-lamp examination revealed corneal filaments in the interpalpebral zone of her left eye (Fig. 2, a). Meanwhile, eyelashes with cylindrical dandruff, lid margin hyperemia and foamy tears were noticed (Fig. 2, b-c). We therefore performed a microscopic examination of the eyelashes and a severe Demodex infestation was confirmed (Fig. 2, d). She denied systemic symptoms such as fatigue, xerostoma, arthralgia, muscular soreness, rashes and loss of appetite or nausea. Combined with her previous diagnosis, we added an additional diagnosis of Demodex blepharitis and long-term lid hygiene and warm compresses were advised. Lid scrub containing a 0.5% solution of tea tree oil twice daily and erythromycin eye ointment once daily were prescribed for Demodex blepharitis. Tobramycin dexamethasone eye ointment was replaced by the relatively low potency fluorometholone 0.02% eye drops. Antibiotic eye drops and artificial tears were continued.

**Fig. 3:**
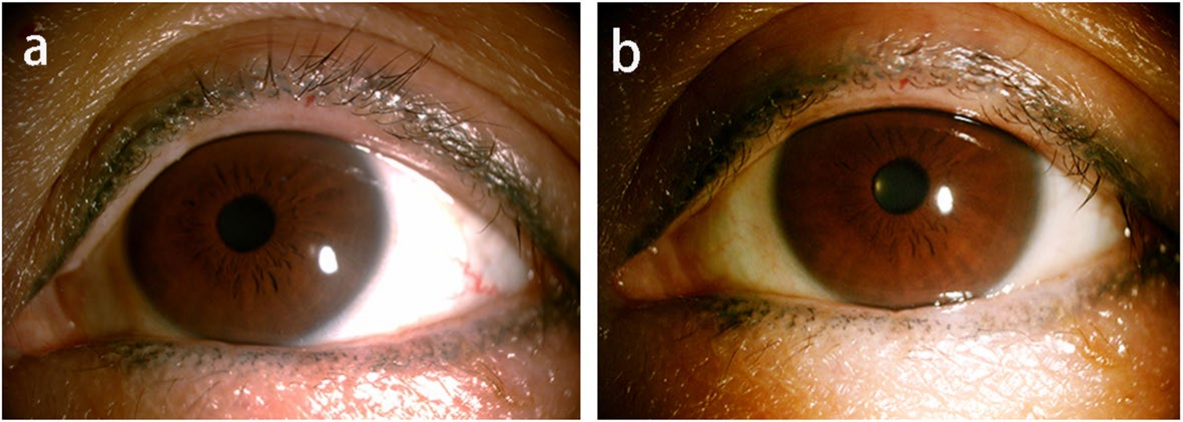
Ocular surface photographs when followed up. **a** no visible filaments 3 weeks after treatment of Demodex infestation. **b** no recurrence a month observed after treatment of Demodex infestation for a year

**Fig. 4:**
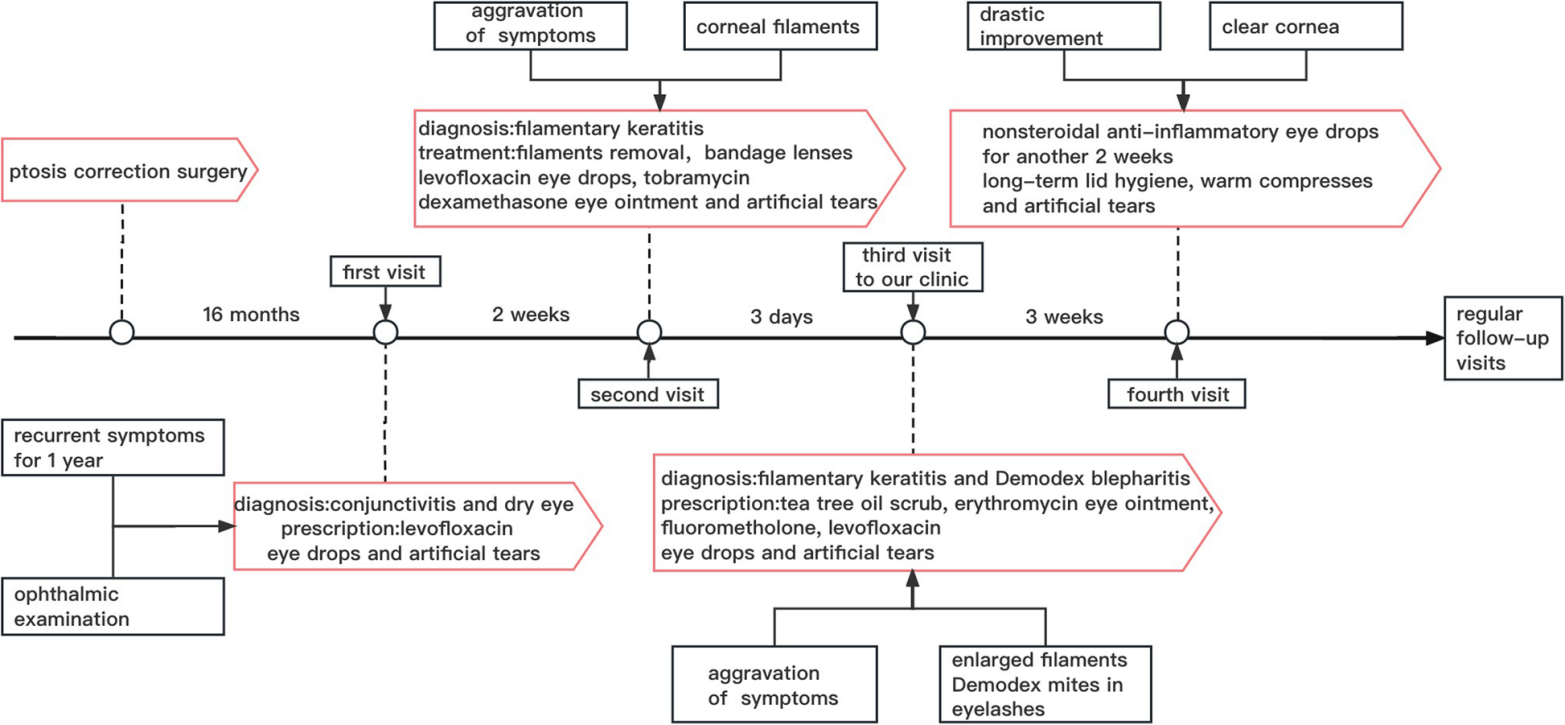
Timeline of the patient’s disease progression and the corresponding treatment

Three weeks later, the patient reported a drastic improvement of her ocular discomfort. Her cornea was clear without filaments (Fig. 3, a) and there was hardly any cylindrical dandruff on her eye lashes. Accordingly, nonsteroidal anti-inflammatory eye drops were applied for another 2 weeks, coming as a substitution for fluorometholone to avoid the long-term adverse effect of topical steroids. In the following 1 year, the patient visited on a monthly basis, presenting a continued symptomatic relief and without signs of filament keratitis (Fig. 3, b). Lid hygiene, warm compresses and preservative-free artificial tears were still recommended to avoid the recurrence of Demodex blepharitis. Timeline of her disease progression is shown in Fig. 4.

## Discussion and conclusions

We present a unique case of refractory filamentary keratitis after a surgical correction of ptosis of the same eye. Surprisingly, our patient did not respond to the conventional treatment and even got worse. Only after the treatment of underlying Demodex infestation did she improve drastically. As we mentioned before, looking for and addressing the underlying etiologies of filamentary keratitis, despite the difficulty, may be the best approach to help patients attain complete remission and prevent recurrence. This case suggested that Demodex infestation, though easily overlooked, may be a risk factor of filamentary keratitis through various mechanisms. Inappropriate medication and ophthalmic operations could even aggravate the progression of filamentary keratitis.

To our knowledge, this is the first report of filamentary keratitis associated with Demodex infestation. Filamentary keratitis is uncommon and considered to be associated with various ocular or systemic conditions, most notably dry eye disease. Ocular Demodex infestation, however, is a very common but highly misdiagnosed condition with an infection rate of 84% of the population at age 60 and in 100% of those older than 70 years [5]. Although some people may present asymptomatic, lots of studies have revealed that Demodex infestation can be a potential risk of ocular inflammation and the prevalence of Demodex mites in patients complaining ocular discomfort is high [6]. When left untreated, Demodex mites are likely to spread and flourish in the eyelids leading to blepharitis. Demodex mites can directly block the orifices of meibomian glands, resulting in meibomian gland dysfunction with lipid tear deficiency. Meanwhile, Demodex mites themselves, their debris or wastes may elicit the host’s inflammatory responses. Inflammation of the lid margin is then spread all over the ocular surface especially in the interpalpebral zone through blinking. This kind of parasite is also confirmed to be a vector of bacteria [7], which further increase the risk of inflammation. All these above may lead to the disturbance of ocular surface microenvironment, promoting corneal lesions and filament formation, which makes Demodex infestation a reasonable cause of filamentary keratitis.

Symptoms of Demodex infestation are non-specific, including eye itching, dryness, irritation and viscous tears, making it easy to overlook these cunning mites as in our case. Demodex blepharitis has long been mis-diagnosed as simply dry eye disease or meibomian gland dysfunction, where the treatment only targets the symptoms and recurrence is usually inevitable [6]. The presence of cylindrical dandruff on the eyelashes is very suggestive of infestation, and parasitological examination of the eyelashes under the microscope can confirm the diagnosis. As for the treatment of Demodex infestation, topical management is more efficient than systemic treatment, among which topical tea tree oil and its active ingredient terpinen-4-ol have achieved good and long-term clinical outcomes [8]. It reminds us to be aware of the involvement of palpebra and think ocular surface as a whole when making a diagnosis. In the overall course of this case, conventional treatment including antibiotic and steroid eye drops failed and her symptoms got even worse. It is a warning of the harmful effects of inappropriate medication which may lead to the progression of filamentary keratitis.

We also notice that her symptoms occurred soon after the surgical correction of ptosis. One study proposed that demodex infestation is a common condition in patients after cataract surgery, the prevalence of which was found to be 48.0% of all studied participants [9]. There is no evidence that Demodex infestation may be related to ophthalmological surgery other than this research. We hypothesize that blepharoplasty or any other ocular surgeries which may interfere with the homeostasis of ocular surface, will make ocular surface vulnerable to Demodex mites or other kinds of infection and inflammation. The disturbance, caused by an ocular surgery, may somehow promote the growth and reproduction of the demodex mites which have already parasitized the host, and continue turning asymptomatic ocular condition into symptomatic diseases, e. g. filamentary keratitis in our case. There are some limitations in this study. Diagnosis, differential diagnosis and evaluations of therapy require detailed clinical examinations, either ocular or systemic. Since systemic diseases such as Sjogren’s Syndrome may lead to ocular manifestations [10], serological examinations are recommended especially when patients have related symptoms. Additionally, this patient did not turn to us until the third visit, so we could only know about her previous ocular surface conditions and examinations results through her medical record, which might lack accuracy to some extent.

In conclusion, our case highlights that the treatment of filamentary keratitis should be tailored to the condition of each patient. It is important to address the underlying cause. Demodex infestation may be related to or can even trigger refractory filamentary keratitis, and it should be taken into consideration when conventional therapy fails to alleviate the symptoms. The role and mechanism of Demodex mites in filamentary keratitis needs to be further investigated.

## Data Availability

Not applicable.

## References

[1] Weiss M, Molina R, Ofoegbuna C (2022). A review of filamentary keratitis. Surv Ophthalmol.

[2] Gumus K, Lee S, Yen MT (2012). Botulinum toxin injection for the management of refractory filamentary keratitis. Arch Ophthalmol.

[3] Hamilton W, Wood TO (1982). Filamentary keratitis. Am J Ophthalmol.

[4] Read SP, Rodriguez M, Dubovy S (2017). Treatment of Refractory Filamentary Keratitis With Autologous Serum Tears. Eye Contact Lens.

[5] Cheng AM, Sheha H, Tseng SC (2015). Recent advances on ocular Demodex infestation. Curr Opin Ophthalmol.

[6] Rabensteiner DF, Aminfar H, Boldin I (2019). Demodex Mite Infestation and its Associations with Tear Film and Ocular Surface Parameters in Patients with Ocular Discomfort. Am J Ophthalmol.

[7] Yan Y, Yao Q, Lu Y (2020). Association Between Demodex Infestation and Ocular Surface Microbiota in Patients With Demodex Blepharitis. Front Med (Lausanne).

[8] Navel V, Mulliez A, Benoist d'Azy C (2019). Efficacy of treatments for Demodex blepharitis: A systematic review and meta-analysis. Ocul Surf.

[9] Nowomiejska K, Lukasik P, Brzozowska A, et al. Prevalence of ocular demodicosis and ocular surface conditions in patients selected for cataract surgery. J Clin Med. 2020;9(10):3069. 10.3390/jcm9103069 PMC7598293 32977656

[10] Roszkowska AM, Oliverio GW, Aragona E, et al. Ophthalmologic manifestations of primary Sjögren's syndrome. Genes (Basel). 2021;12(3):365. 10.3390/genes12030365 PMC7998625 33806489

